# Case Report: Sapho syndrome complicated by chest pain successfully treated with relocation

**DOI:** 10.3389/fimmu.2026.1776452

**Published:** 2026-06-02

**Authors:** Youjun Zhang, Hongjin Chen, Shaofeng Guan, Xinkai Qu

**Affiliations:** 1 Department of Cardiology, Huadong Hospital, Fudan University, Shanghai, China; 2 Shanghai Key Laboratory of Clinical Geriatric Medicine, Shanghai, China

**Keywords:** case report, chest pain, environmental factor, relocation, SAPHO syndrome

## Abstract

Synovitis, acne, pustulosis, hyperostosis, and osteitis (SAPHO) syndrome is a rare chronic autoinflammatory disease characterized primarily by cutaneous manifestations and osteoarticular involvement, and remains challenging to treat. Its pathogenesis is not fully understood and may involve genetic, immunological and environmental factors. Environmental factors play a significant role in autoimmune diseases such as asthma, urticaria, and rheumatic heart disease. Avoiding repeated exposure to sensitizing environmental trigger (e.g., microorganism or allergens) shows considerable potential in the treatment of above immune mediated disorders. This article reports a case of a 60-year-old female admitted with recurrent chest pain for over two months accompanied by periodic plantar pustules. A diagnosis of SAPHO syndrome was made based on her medical history, clinical manifestations, and bull’s head sign on bone scintigraphy findings, after excluding other potential causes. Notably, the patient’s husband had ankylosing spondylitis, and neither the patient nor her husband had a family history of inherited immune diseases. This rare occurrence in cohabiting spouse suggests that they shared environmental exposures may contribute to the development of phenotypically overlapping immune diseases. Therefore, we advised her to try changing her residential environment (moving to a drier, better ventilated environment) and iodophor disinfection of soles. Subsequently, her plantar pustules and chest pain were significantly relieved and remained relapse free. We first reported a case of successful environmental modification as an adjunctive treatment for SAPHO syndrome, suggesting a potential role of environmental factors in pathogenesis and clinical management of this condition.

## Introduction and case description

SAPHO syndrome is a rare chronic autoinflammatory disease. Currently, there are no validated diagnostic criteria for SAPHO syndrome, thus diagnosis is primarily based on clinical features and imaging findings. The main challenge in diagnosis lies in the fact that its clinical features may manifest years apart or may not even present all typical manifestations. SAPHO syndrome is typically characterized by a bull’s head sign on bone scintigraphy findings, along with skin manifestations including palmoplantar pustulosis or severe acne. The exact etiology and pathogenesis of SAPHO syndrome remain unclear, but it may involve a combination of genetic, infectious, and immunological factors that trigger the activation of the autoimmune system. One pathogenic hypothesis suggests that propionibacterium acnes may trigger autoimmune dysregulation in genetically susceptible individuals (1), may involve the abnormal activation of the interleukin (IL)-1β, tumor necrosis factor-α (TNF-α), and IL-17 pathways (2–5). Current treatments primarily focus on inhibiting inflammation and modulating immunity, including the use of nonsteroidal anti-inflammatory drugs (NSAIDs), steroids. Novel biologics and targeted small molecules, such as JAK inhibitors, have been reported to be effective in some cases (6, 7). Avoiding exposure to environmental triggers such as pathogenic microorganisms or allergens may be a novel complementary solution in SAPHO syndrome. Here, we report a case of SAPHO syndrome that improved after changing the living environment (moving from a damp environment to a drier, better ventilated one). We will discuss the possible mechanisms and therapeutic insights of changing the living environment in this disease.

**Figure 1:**
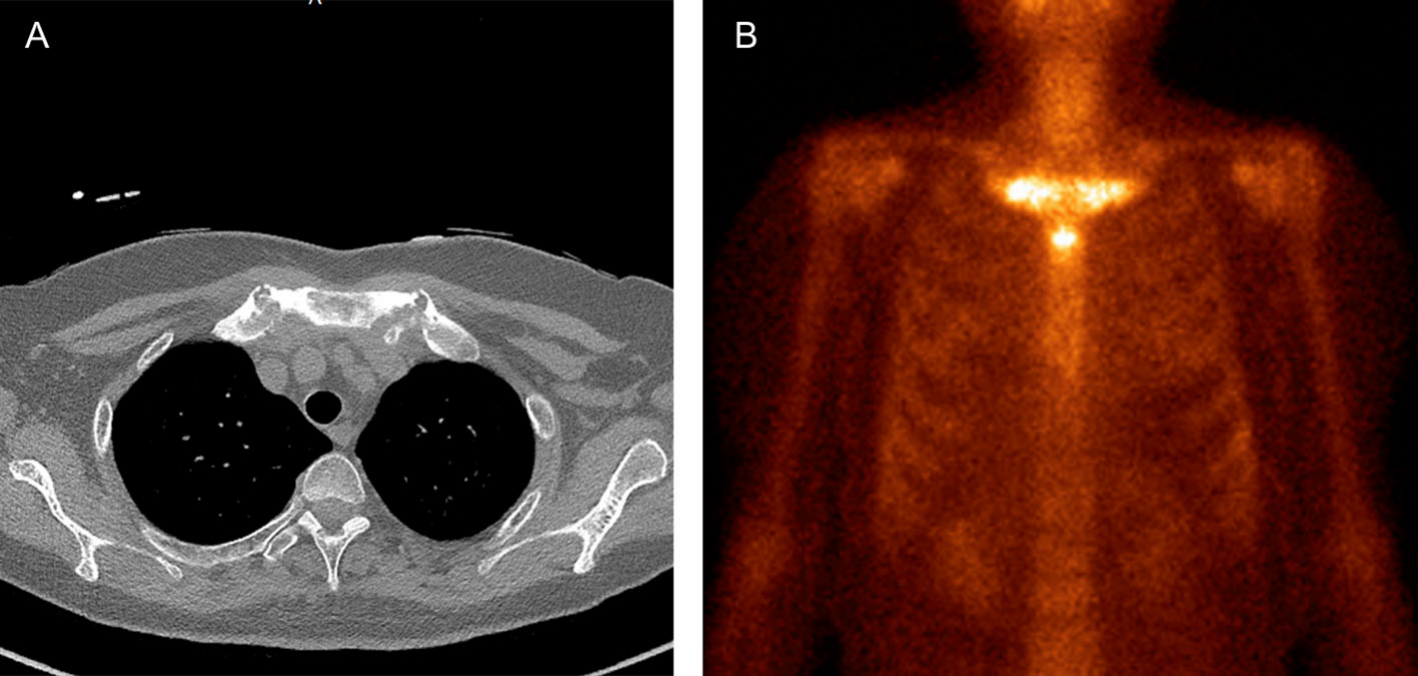
**(A)** Physical examination revealed multiple pustules on both soles with mildly itchy. **(B)** Multiple pustules on soles gradually improved and significantly relieved with 2-month relocation and iodophor disinfection.

**Figure 2:**
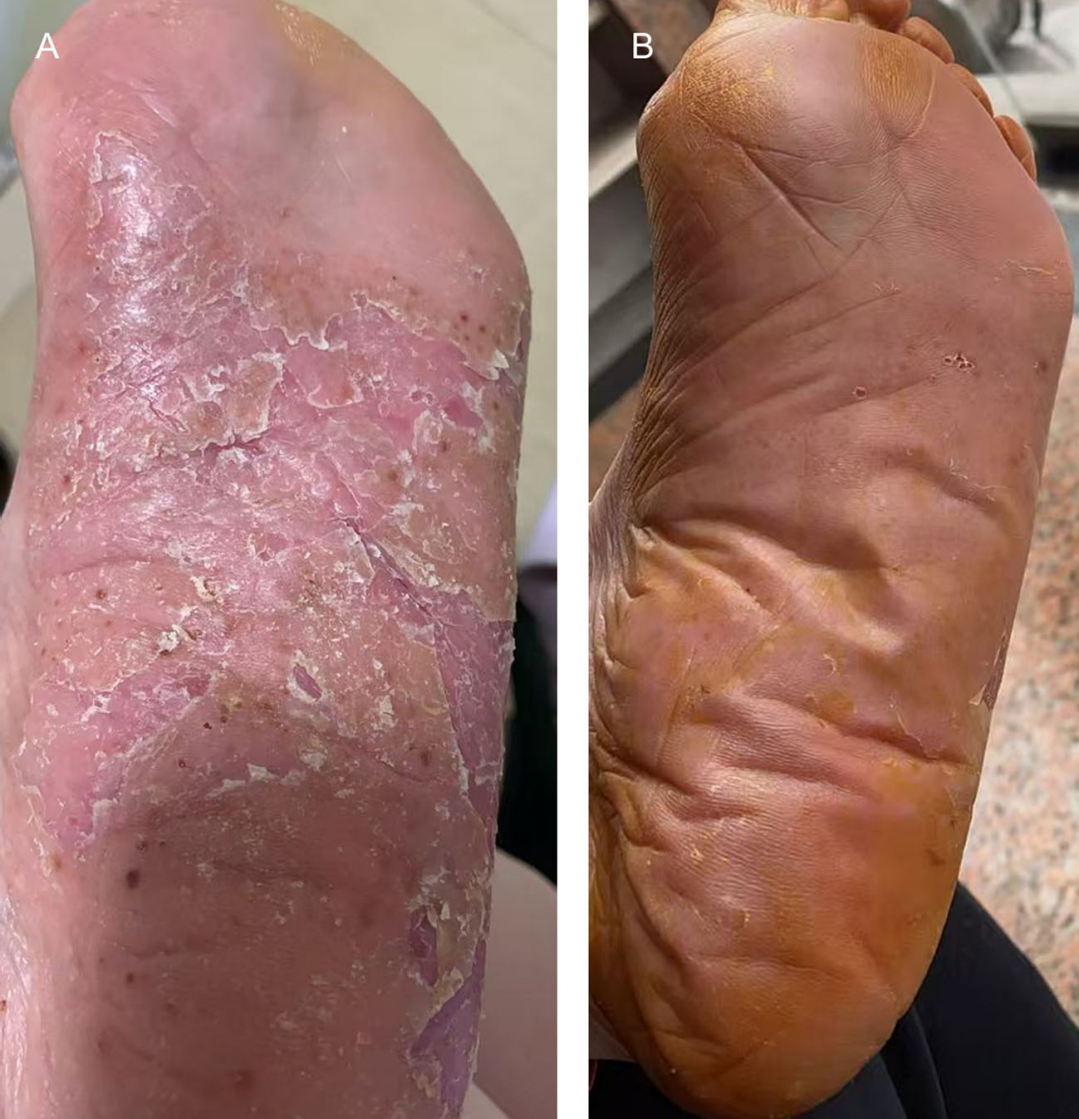
**(A)** Chest CT showing significant sternocostoclavicular hyperostosis and bone density increased. **(B)** Bone scintigraphy shows the bull’s head sign: osteosclerosis of the bilateral first costosternal joints and manubriosternal joint with markedly increased bone metabolic activity.

A 60-year-old woman with a 2-month history of chest pain was referred to our cardiac department. The pain, located in the mid-chest and radiating to both shoulders, was progressive and worsened with movement. She also reported pustular lesions on both soles, accompanied by mild pruritus, recurring cyclically for one year. Physical examination revealed multiple mildly pruritic pustules on both soles (**Figure 1A**). Laboratory evaluations, including complete blood cell count, routine biochemical tests, troponin and C-reactive protein levels, and erythrocyte sedimentation rate, were within normal limits. Coronary computed tomography angiography (CTA) and electrocardiography also revealed no abnormalities. Chest computed tomography (CT) revealed increased bone density of the sternal manubrium and the medullary cavity of the sternal body (**Figure 2A**). Whole body scintigraphy revealed increased radiotracer uptake in the sternoclavicular, manubriosternal, and sacroiliac joints. The increased [99mTc]Tc-MDP uptake in the manubriosternal and both sternoclavicular regions represents the bull’s head sign, which is a rare finding, but indicative of the diagnosis of SAPHO (**Figure 2B**) (8). Further inquiry revealed no significant personal medical history, trauma, or remarkable family history, but the patient’s husband was diagnosed with HLA-B27 negative ankylosing spondylitis several years ago. Although they are not biologically related, both individuals were affected by diseases that share some phenotypic similarities. This rare occurrence in cohabiting spouse suggests that they shared environmental exposures may contribute to the development of phenotypically overlapping immune diseases. She further reported living in a damp, ground floor residence. After excluding the possibilities of other causes, treatment with corticosteroids and/or NSAIDs was advised. However, the patient refused due to concerns regarding potential side effects and the necessity for long term administration. Consequently, we advised her to try relocating to a drier, well ventilated residence on a higher floor, combined with a topical corticosteroid ointment to the soles and disinfection with iodophor per day. Interestingly, at a follow up examination two months after relocation, the patient reported the chest pain was almost disappeared for two weeks, and the plantar pustules responded poorly to steroid ointment in the first two weeks, but gradually improved and significantly relieved following the addition of iodophor disinfection for more three weeks (**Figure 1B**). During the subsequent five months follow up, relapse of plantar pustules and chest pain were not observed.

## Discussion

SAPHO syndrome is a rare clinical condition characterized by osteoarticular and cutaneous manifestations. The anterior chest wall is involved in up to 90% of cases, followed by the sacroiliac joints and spine (30-35%) (9). Palmoplantar pustulosis is present in 55-65% of patients, whereas severe acne is observed in approximately 25% (10).

To date, validated diagnostic criteria for SAPHO syndrome are lacking; therefore, diagnosis relies primarily on clinical findings and characteristic radiological changes, with or without elevated inflammatory markers. Osteoarticular and cutaneous manifestations often do not occur synchronously and lack a clear temporal association, complicating early diagnosis (11, 12), as illustrated in this case. In patients with anterior chest wall involvement, chest CT can be an important screening tool. CT can reveal cortical bone erosion, diminished joint space, ligamentous ossification, periosteal proliferation, and sclerosis. Bone scintigraphy is a valuable diagnostic tool. The typical bull’s head sign on bone scintigraphy is highly suggestive of SAPHO syndrome (13), as shown in this case (**Figure 2B**). In addition, carefully excluding the possibility of psoriasis, infectious causes, osteoarthritis, coronary artery disease, eczema and malignant diseases is necessary (Supplementary Material) (14).

The etiology and pathogenesis of SAPHO syndrome are not fully understood but are thought to involve an interplay of infectious, immunological, and genetic factors, leading to dysregulation of the innate and cellular immune systems (15). Propionibacterium acnes has been isolated from biopsy specimens of many patients with SAPHO syndrome (2). Since P. acnes is a component of the normal cutaneous flora, it is often neglected as a contaminant (16). In our patient, which seems to be a sign of spousal aggregation, changing the living environment appeared to have a beneficial effect on the disease, and inspired by the immunopathological mechanisms of diseases such as rheumatic heart disease, asthma, and urticaria, therefore we speculated that the original living environment may have harbored specific microorganisms or allergens (may not necessarily P. acnes) capable of triggering the clinical manifestations of SAPHO syndrome in this patient. In recent years, growing evidences have highlighted the pivotal role of environmental triggers, such as viruses, fungus, bacteria, dust mite and even stress, in influencing immune balance and trigger autoimmunity (17, 18). Mechanistically, in genetically susceptible populations, microorganisms can trigger autoimmune responses through molecular mimicry and cross reactivity, such as rheumatic heart disease (19, 20). Allergens can induce asthma by promoting Th2 cell activation (21). Similarly, allergens can activate B cells and mast cells to trigger urticaria (22). In different autoimmune diseases, the predominant inflammatory cell (e.g., neutrophils, T cells, eosinophils, and mast cells) and cytokines (e.g., TNF-α, IL-1, and IL-17) may vary, but a unifying feature is the involvement of environmental triggers. Therefore, we draw an inference that avoidance of potential sources of environmental triggers could represent a novel therapeutic approach for some patients with SAPHO syndrome, as suggested by this case. This aligns with earlier studies (23), which found that the efficacy of antibiotic therapy for SAPHO syndrome is lost after its discontinuation, which may be explained by the suppression of certain microorganisms during treatment, followed by re-exposure to the same pathogens from the patient’s specific environment after antibiotics are stopped. Moreover, early detection and implementation of such avoidance strategies may lead to better outcomes, as prolonged microbial colonization can persistently stimulate the immune system. Once such colonization is established, reducing exposure may be less effective. On the other hand, multiple studies have attempted to identify cytokines, such as TNF-α, IL-1, and IL-17, as immunological contributors to the chronic inflammation in SAPHO syndrome (24). However, none of these associations have been conclusively confirmed (25). Other immune molecules likely involved require further investigation. Furthermore, genes within the human leukocyte antigen (HLA) family have been implicated as potential genetic factors in SAPHO, although none have been definitively validated (26). Investigations to identify the specific genes involved are still ongoing.

The specific pharmacotherapy suitable for SAPHO syndrome remains unclear. The primary goals of treatment are to relieve pain and control inflammation. NSAIDs and corticosteroids are the mainstay of therapy. Osteoarticular pain can be alleviated to some extent with anti-inflammatory therapy. Recent studies have also reported some biologic agents (e.g., TNF inhibitors), targeted small molecules (e.g., JAK inhibitors) and disease modifying antirheumatic drugs (DMARDs) have achieved therapeutic effects (7, 27, 28). Cutaneous manifestations may be treated with topical corticosteroids, although the response is often suboptimal, as shown in this case. The disease course is often protracted, spanning several years with recurrent episodes of remission and relapse (2). Nevertheless, the long term prognosis is generally favorable. The long term systemic use of anti-inflammatory agents requires careful balancing of benefits and risks. In the present case, avoidance of potential triggers (e.g., infectious agents or allergens) could be considered a form of adjunctive therapy. Collectively, for such patients, temporary targeted anti-inflammatory treatment combined with long term exposure reduction, including environmental modification and iodophor disinfection, may be a preferable management strategy.

Since this is a single case report, which has limitations due to a relatively short follow up period, lacking of immunological profiling, and insufficient objective outcome measures. The concept of SAPHO syndrome as a reactive osteitis to environmental triggers seems appealing, but yet to be confirmed and further studies are warranted.

## Data Availability

The original contributions presented in the study are included in the article/supplementary material. Further inquiries can be directed to the corresponding author.
